# Arthroscopic Resection of Intra-articular Fibroma of a Tendon Sheath of the Knee: A Case Report

**DOI:** 10.7759/cureus.105784

**Published:** 2026-03-24

**Authors:** Hidefumi Suzuki, Naoya Kikuchi, Hiroshi Akaogi, Kazuhiro Ikeda

**Affiliations:** 1 Department of Orthopaedic Surgery, Tsukuba Memorial Hospital, Tsukuba, JPN; 2 Department of Orthopaedic Surgery, Faculty of Medicine, University of Tsukuba, Tsukuba, JPN; 3 Orthopaedic Surgery, Akaogi Orthopaedic Clinic, Tsukuba, JPN

**Keywords:** arthroscopy, fibroma of the tendon sheath, knee joint, posterior compartment, tumor

## Abstract

Fibroma of the tendon sheath (FTS) is a benign fibrous tumor arising from the synovium of the tendon sheath and only rarely presents as an intra-articular lesion in the knee. We present a case of intra-articular FTS of the knee, successfully treated with arthroscopic excision. A 48-year-old woman presented with gradually progressive posterior knee pain for a year. On examination, the knee range of motion was limited to 0-120°, with pain elicited at both extension and flexion. Magnetic resonance imaging (MRI) demonstrated a well-defined intra-articular mass with a size of 25 × 20 × 15 mm, located posterior to the posterior cruciate ligament (PCL). The lesion was homogeneously hypointense on T1-weighted images and isointense on T2-weighted images, clearly separated from the surrounding structures. Based on these findings, an intra-articular benign tumor was suspected. Arthroscopic tumor excision was performed through a posterolateral portal. The mass was identified posterior to the PCL and resected en bloc. Histopathological examination revealed a nodular lesion composed of densely proliferating spindle-shaped cells within a collagen-rich stroma, with focal hyalinization. No cellular atypia or mitotic figures were observed. Neither multinucleated giant cells nor hemosiderin deposits were identified. Postoperatively, the patient’s range of motion improved, and no recurrence has been observed at one-year follow-up. Arthroscopic excision of intra-articular FTS of the knee appears to be a safe and effective treatment. FTS should be considered in the differential diagnosis of intra-articular knee tumors. Arthroscopic excision represents a safe and effective option in such rare cases.

## Introduction

Fibroma of the tendon sheath (FTS) is a benign fibrous tumor that arises from the synovial lining of tendon sheaths. It most frequently occurs in the fingers and wrists, accounting for most reported cases, whereas involvement of large joints is rare [1].

In the large series of 138 cases, 86% of FTS occurred typically in young to middle-aged men in the upper extremity - most commonly along the flexor tendons of the fingers (49%), followed by the palm (21%), wrist (12%), forearm (1%), elbow (2%), and arm (1%). Lesions in the lower extremity accounted for only 14% [2]. FTS arising around large joints (knees, elbows, shoulders, and ankles) is rare. FTS in the knee particularly originates from the joint capsule. These intra-articular tumors are typically found in the posterior compartment, often adjacent to the PCL or posterior capsule [3,4]. In a review of FTS around large joints, the mean age of patients was 40.9 years (range 13-69), and about 60% were male. The predominant symptoms were pain (62.5%), swelling or palpable mass (54.2%), and limited range of motion (50%). A history of knee trauma was reported in approximately 10% of the cases [3]. Histologically, FTS is characterized by a nodular or lobulated architecture composed of bland spindle-shaped fibroblast-like cells embedded in a dense collagenous stroma, often containing elongated slit-like vascular spaces [5].

FTS may mimic other benign or malignant intra-articular lesions, including giant cell tumor of the tendon sheath (GCTTS), nodular fasciitis (NF), and synovial sarcoma (SS) [6]. Accurate differentiation among these entities is clinically important because their biological behavior, treatment strategies, and prognoses differ substantially. For example, GCTTS may show locally aggressive behavior with a higher risk of recurrence, NF may exhibit rapid growth that can raise concern for malignancy, and SS represents a malignant tumor requiring wide resection and oncologic management. Therefore, careful evaluation using imaging, intraoperative findings, and histopathological examination is essential for establishing the correct diagnosis.

Although several cases of FTS around large joints have been reported, intra-articular FTS of the knee remains rare. In particular, only a limited number of cases describing arthroscopic management have been reported in the literature. Especially in the posterior compartment, surgical access can be technically demanding, and the optimal surgical approach has not been fully established.

We encountered a case of intra-articular FTS of the knee successfully treated by arthroscopic resection and discuss the clinical and pathological features with reference to the relevant literature.

## Case presentation

A 48-year-old woman presented with a one-year history of gradually progressive posterior pain in the right knee. She reported no history of trauma, but had a past medical history of atopic dermatitis and cutaneous sarcoidosis. The patient was evaluated and treated at Tsukuba Memorial Hospital, Tsukuba, Ibaraki, Japan.

On physical examination, the range of motion was restricted to 0-120°, with pain at both extension and flexion. No visible swelling, effusion, or palpable mass was noted. Laboratory tests, including inflammatory markers, were within normal limits (Table 1). The absence of inflammatory findings, together with slowly progressive posterior knee pain, suggested a benign intra-articular process rather than inflammatory or degenerative pathology.

**Table 1:** Initial laboratory investigations with their reference ranges. Laboratory tests were within normal limits.

Lab test	Result	Reference range
White blood cell count	5.1 ×10^9/L	3.5–9.0 × 10^9^/L
Hemoglobin	11.6 g/dL	11.3–15.2 g/dL
Platelet count	200 ×10^9/L	150–350 × 10^9^/L
C-reactive protein	<0.03 mg/dL	<0.3 mg/dL
Albumin	4.1 g/dL	3.8–5.3 g/dL
Total protein	6.7 g/dL	6.6–8.1 g/dL
Creatinine	0.53 mg/dL	0.6–1.1 mg/dL
Blood urea nitrogen	10 mg/dL	8–20 mg/dL
Estimated glomerular filtration rate (eGFR)	94.5 mL/min/1.73m²	≥60 mL/min/1.73 m²
Sodium	141 mmol/L	138–145 mmol/L
Potassium	4.2 mmol/L	3.6–4.8 mmol/L
Chloride	106 mmol/L	101–108 mmol/L
Aspartate aminotransferase	22 U/L	13–30 U/L
Alanine aminotransferase	15 U/L	10–42 U/L
Total bilirubin	0.5 mg/dL	0.4–1.5 mg/dL
Glucose	93 mg/dL	70–109 (fasting) mg/dL
Hemoglobin A1c (HbA1c)	5.1 %	4.6–6.2%

**Figure 1:**
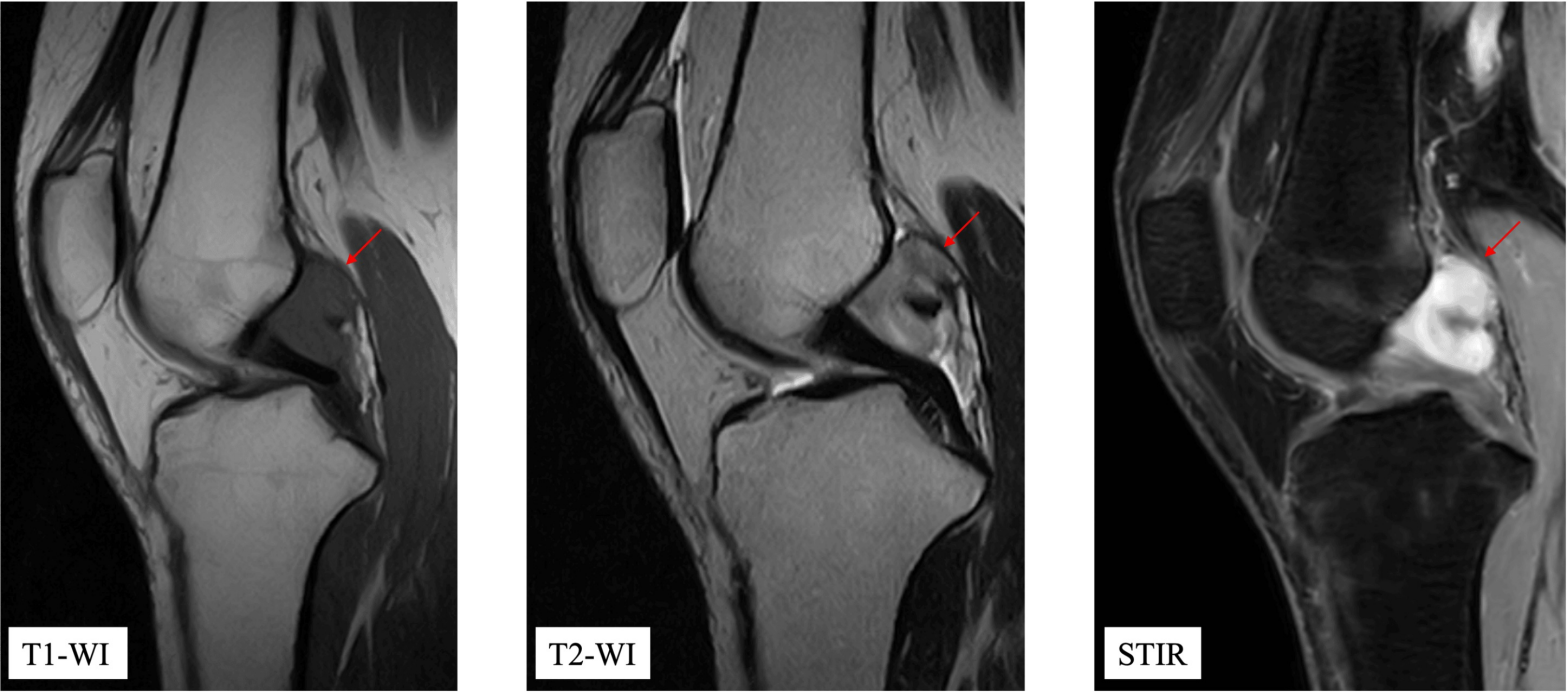
Preoperative MRI of the right knee. T1-weighted, T2-weighted, and STIR images demonstrate a well-defined intra-articular mass located posterior to the femoral attachment of the posterior cruciate ligament. The lesion is hypointense on T1-weighted images and isointense on T2-weighted images, without surrounding bone marrow edema or joint effusion.

MRI revealed a well-demarcated intra-articular mass measuring 25 × 20 × 15 mm, located posterior to the femoral attachment of the PCL (Figure 1).

**Figure 2:**
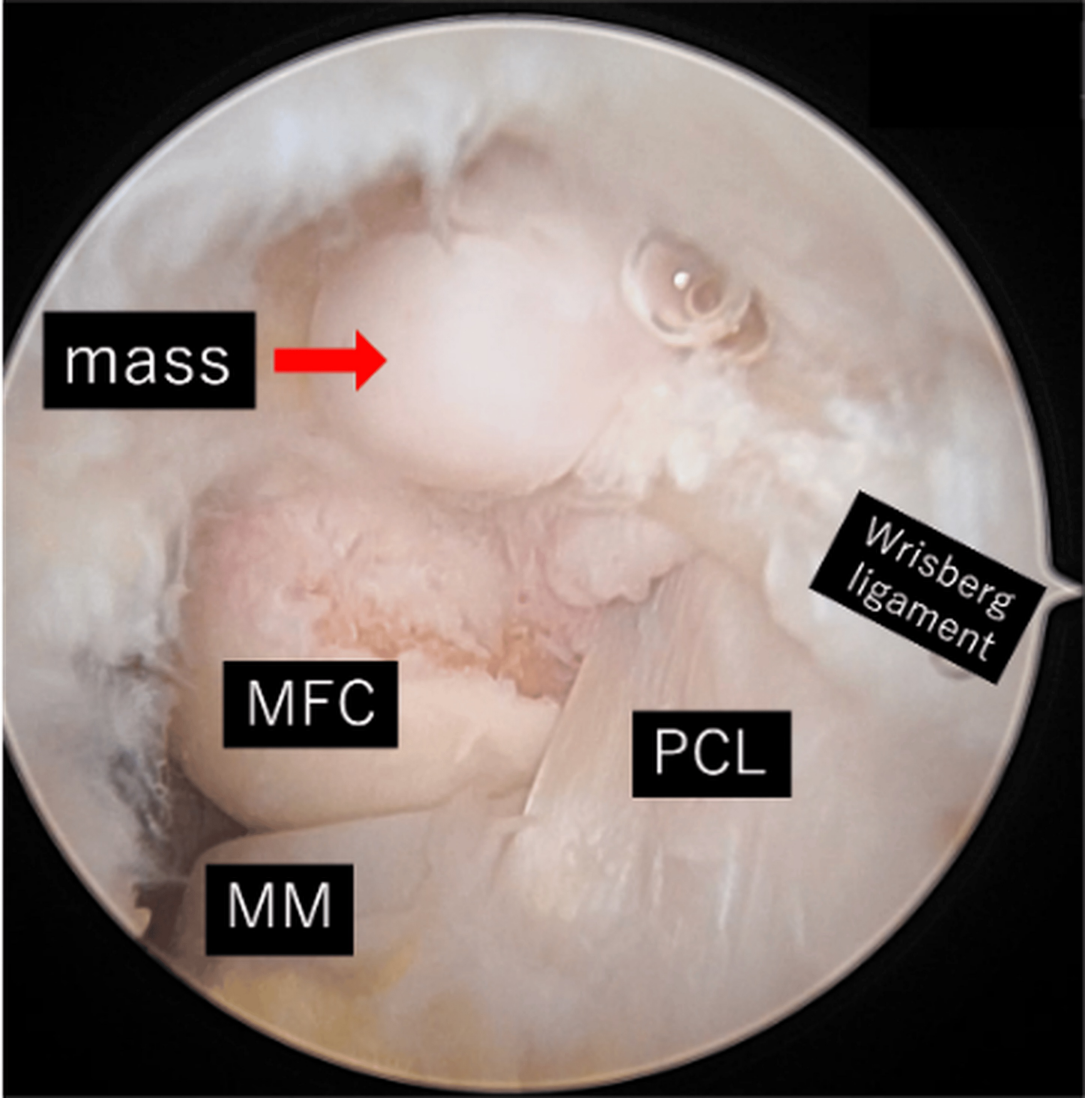
Intra-articular view by arthroscopy. A mass was identified posterior to the PCL, clearly separated from the adjacent synovium and ligament. PCL; posterior cruciate ligament, MFC; medial femoral condyle, MM; medial meniscus

The lesion was homogeneously hypointense on T1-weighted images and isointense on T2-weighted images, without surrounding bone marrow edema or joint effusion. No enhancement was observed outside the lesion margin. Based on the slowly progressive clinical course, absence of inflammatory laboratory abnormalities, and MRI findings of a well-defined intra-articular mass without invasive features, a benign intra-articular soft-tissue tumor was considered most likely preoperatively. Arthroscopic resection was performed using a posterolateral portal. Intraoperatively, a firm, nodular, whitish mass was identified posterior to the PCL, clearly separated from the adjacent synovium and ligament (Figure 2).

**Figure 3:**
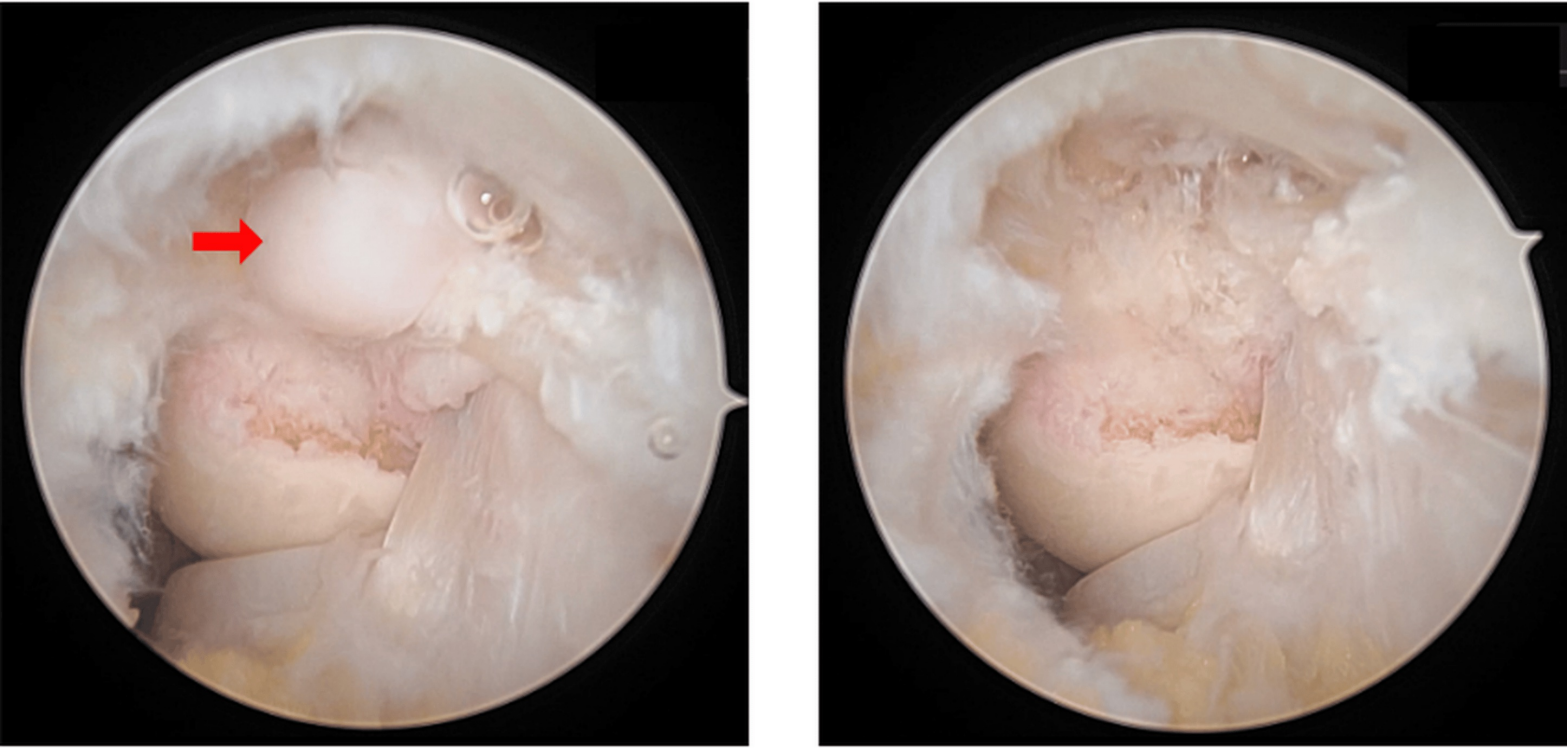
Posterolateral view of before (left) and after (right) arthroscopic resection.

**Figure 4:**
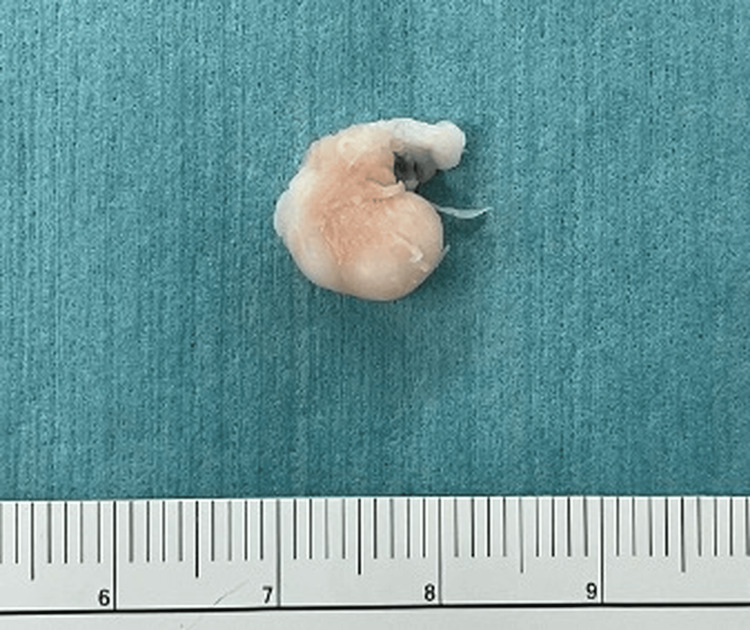
The tumor excised through posterolateral portal.

The tumor was completely excised en bloc by arthroscopy without complications (Figures 3, 4).

**Figure 5:**
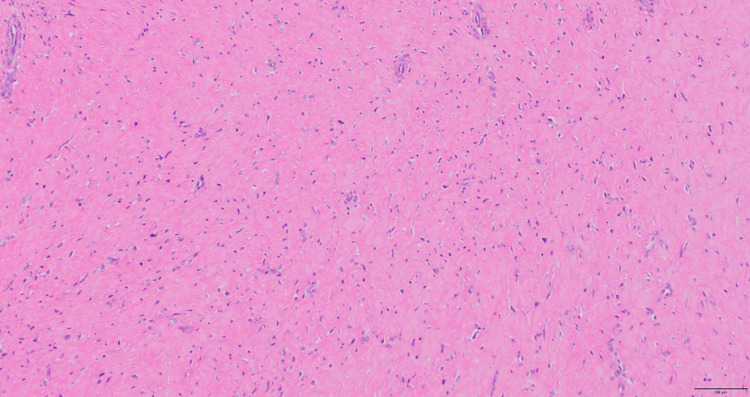
Hematoxylin and eosin stain (H&E)-stained section of the mass. A nodular lesion composed of densely proliferating spindle-shaped cells within a collagen-rich stroma was observed, with areas of hyalinization.

Histopathological examination in hematoxylin and eosin staining revealed a nodular lesion composed of densely proliferating spindle-shaped cells within a collagen-rich stroma, with areas of hyalinization. No mitotic figures, nuclear atypia, multinucleated giant cells, or hemosiderin deposition were observed (Figure 5).

These features were consistent with FTS and distinguished the lesion from GCTTS [6].

The patient’s postoperative course was uneventful. Range of motion exercises were initiated on the first postoperative day, and weight-bearing was permitted as tolerated. Knee pain resolved progressively, and a full range of motion was achieved within two months. At one-year follow-up, the patient had returned to unrestricted daily activities without pain, and follow-up MRI demonstrated no evidence of recurrence.

All images are original clinical and intraoperative photographs from the patient described in this report. Written informed consent for publication was obtained.

## Discussion

FTS is a relatively common benign lesion of the hand, but is exceedingly rare in large joints. A systematic review identified 43 cases of FTS arising around large joints, with 74% in the knee [3]. FTS typically presents as a “slowly growing, painless mass,” but in the knee, it is often associated with pain, swelling, or motion restriction. The mean age was in the fourth decade, with a slight male predominance. Intra-articular lesions most commonly arise in the knee, particularly in the posterior compartment adjacent to the posterior cruciate ligament or posterior capsule [3]. Although less common, involvement of other large joints such as the ankle and shoulder has also been reported. Most cases have been treated with marginal resection using either open or arthroscopic approaches, and no recurrences have been reported following complete excision. Our patient’s demographic characteristics and clinical presentation, including middle age at onset with posterior knee pain and flexion restriction, are consistent with previous reports [2,3]. In comparison with previously reported intra-articular FTS cases, the present case demonstrated typical features such as posterior compartment localization and mechanical symptoms in the absence of inflammatory findings. These observations support the recognition of intra-articular FTS as a rare but clinically identifiable entity.

Imaging characteristics

MRI is a useful tool but not diagnostic. FTS typically shows low signal on T1-weighted images and variable intensity on T2-weighted images, depending on the proportion of collagenous, hyalinized, and myxoid components [7]. The iso-intensity on T2-weighted images in the present case may be explained by the histological composition: abundant collagenous and hyalinized stroma, which usually leads to low signal, combined with areas containing more hydrated or myxoid components, resulting in an overall signal intensity similar to that of muscle. Although FTS generally shows low signal intensity on all MRI sequences, some cases demonstrate variable signal patterns; therefore, correlation with histopathological findings is required for accurate diagnosis [8]. Intra-articular FTS often demonstrates well-defined margins without adjacent bone changes, which may help differentiate it from aggressive lesions. However, overlap exists with GCTTS and NF. GCTTS often demonstrates blooming artifacts on gradient-echo sequences due to hemosiderin, while FTS lacks this feature [9].

Histopathology

Histological confirmation is essential for diagnosis. FTS is characterized by bland spindle cells embedded in dense collagen with slit-like vascular channels, without atypia or hemosiderin [10]. These features help distinguish FTS from GCTTS, which contains abundant multinucleated giant cells and hemosiderin, and from NF, which demonstrates myxoid stroma and mitotic activity [2,10].

Molecular and cytogenetic findings

Cytogenetic and molecular genetic investigations have demonstrated that fibroma of the tendon sheath (FTS) is a true neoplastic lesion characterized by non-random chromosomal rearrangements. The previous report described clonal karyotypic alterations such as t(2;11)(q31;q12) [11]. Recent analyses have identified recurrent USP6 gene fusions (e.g., USP6-MYH9, USP6-COL1A1, USP6-ASPN) in both classic and cellular variants of FTS [12]. These findings support the concept that FTS represents a benign fibroblastic/myofibroblastic neoplasm with characteristic cytogenetic and molecular profiles rather than a reactive proliferation.

Treatment and prognosis

The standard treatment is complete surgical excision. Arthroscopic techniques have gained favor for intra-articular lesions, especially those adjacent to the PCL and posterior capsule, as they allow safe and minimally invasive access [3,6]. In the present case, a posterolateral portal was selected to access the posterior compartment because the lesion was located immediately posterior to the posterior cruciate ligament (PCL). Arthroscopic access to this region can be technically demanding due to limited visualization and the risk of injury to the popliteal neurovascular structures. The posterolateral portal provided direct visualization of the lesion and allowed safe instrument manipulation while minimizing the risk of neurovascular injury. This approach enabled complete en bloc resection under adequate visualization and preserved surrounding synovial and ligamentous structures. Several authors have reported favorable outcomes with arthroscopic resection, with minimal recurrence when complete removal is achieved [3,6,7,10,13]. A previous report suggested a recurrence rate of up to 24%, but these were largely from hand lesions; recurrence in intra-articular large-joint cases appears to be rare [3]. In our case, arthroscopic en bloc excision achieved excellent functional recovery and no recurrence at one year.

Clinical implications: Clinically, intra-articular FTS should be suspected in patients presenting with slowly progressive knee pain, mechanical motion limitation, and a well-defined posterior intra-articular mass without inflammatory laboratory abnormalities.

The differential diagnosis of intra-articular FTS includes GCTTS, NF, and SS (Table 2) [14-17].

**Table 2:** Differential diagnosis of intra-articular fibroma of the tendon sheath (FTS) .

Feature	FTS (fibroma of the tendon sheath)	GCTTS (giant cell tumor of the tendon sheath)	NF (nodular fasciitis)	SS (synovial sarcoma)
Pathologic type	Benign fibrous neoplasm of the tendon sheath	Benign proliferative lesion with histiocytic and giant cells	Benign, self-limited myofibroblastic proliferation	Malignant mesenchymal neoplasm with epithelial differentiation
Typical location	Fingers, wrist; rarely large joints (knee, ankle)	Fingers, hand, ankle	Upper extremity, trunk, and sometimes knee	Periarticular or intra-articular regions (knee, foot, hip)
Clinical presentation	Slowly growing, usually painless nodule	Slow-growing, may be painful	Rapidly growing, sometimes tender	Deep-seated mass, pain, or swelling common
MRI findings	Well-defined, uniformly low T1 / T2; minimal or no enhancement	Low T1, variable T2 with blooming (hemosiderin); strong enhancement	Iso- to high T2 with myxoid areas; variable enhancement	Heterogeneous solid or myxoid lesion; strong enhancement; may contain hemorrhage
Histopathology	Bland spindle cells in dense collagenous stroma with slit-like vessels	Multinucleated giant cells, hemosiderin deposition, and histiocytes	Myxoid stroma, tissue-culture–like fibroblasts, frequent mitoses	Biphasic or monophasic spindle cells; epithelial elements may be present
Immunohistochemistry	Vimentin+, SMA±, Cytokeratin–, S-100–	CD68+, CD163+, SMA±	SMA+, S-100–, Desmin−	Cytokeratin+, EMA+, bcl-2+, TLE1+, S-100–
Genetic / Molecular findings	USP6 gene fusions (e.g., USP6–MYH9, USP6–COL1A1)	No specific fusion	USP6 rearrangement (often MYH9–USP6)	SYT-SSX fusion (t(X;18)(p11;q11))
Biologic behavior / Prognosis	Benign; rare recurrence after complete excision	Benign; local recurrence possible	Self-limited; spontaneous regression possible	Malignant; local recurrence and metastasis possible
Treatment	Marginal or arthroscopic excision	Marginal excision	Simple excision or observation	Wide or extra-articular resection ± chemo/radiotherapy

GCTTS is the most frequent radiologic mimic, as both lesions arise adjacent to the tendon sheath and may present with pain and limited motion [3]. However, GCTTS typically exhibits more pronounced contrast enhancement and may cause cortical pressure erosion due to hemosiderin deposition and multinucleated giant cells, while FTS demonstrates uniformly low signal intensity on all MRI sequences with minimal enhancement and rarely induces bony change. Minimal or absent contrast enhancement is more suggestive of FTS [15].

NF is a benign, self-limiting myofibroblastic proliferation that can mimic malignant tumors such as low-grade SS because of its rapid growth, high cellularity, and mitotic activity [16]. Compared with FTS, NF shows a less organized, tissue culture-like growth pattern, more prominent myxoid stroma, extravasated red blood cells, and focal inflammatory infiltration, while lacking the dense collagen bundles and well-organized vascular pattern typical of FTS. NF is usually α-SMA positive and S-100 negative, and detection of a USP6 gene rearrangement (often MYH9-USP6 fusion) supports the diagnosis. It generally follows a benign course and may regress spontaneously without aggressive resection.

SS arising intra-articularly is extremely rare but should be considered in the differential diagnosis of soft-tissue masses within the knee joint [17]. Unlike FTS, SS typically presents as a solid or partially cystic/myxoid mass with heterogeneous signal intensity on MRI, often showing marked contrast enhancement and possible hemorrhagic areas. Histologically, SS exhibits biphasic or monophasic spindle cell patterns, with immunoreactivity for cytokeratin, EMA, and bcl-2, and is characterized by the SYT-SSX gene fusion. SS behaves as a malignant tumor with a risk of local recurrence or metastasis, often requiring wide or extra-articular resection, whereas FTS is benign and cured by local excision.

Recognition of FTS as a slowly progressive, well-defined nodule attached to the tendon sheath is crucial to avoid misdiagnosis and unnecessary wide resection. Arthroscopic marginal excision not only allows histologic confirmation but also provides complete resection and excellent postoperative recovery without recurrence.

This study has limitations inherent to a single case report, including limited generalizability and a relatively short follow-up period. Longer-term follow-up and additional cases are required to further clarify recurrence risk and long-term outcomes.

## Conclusions

Intra-articular FTS of the knee is an extremely rare entity that should be considered in the differential diagnosis of benign intra-articular lesions. Although histopathological examination remains essential for definitive diagnosis, arthroscopic excision appears to be a safe and effective treatment option in selected cases. Further case accumulation and longer follow-up are needed to clarify long-term outcomes and recurrence risk.
